# Giant lactating adenoma

**DOI:** 10.4322/acr.2021.252

**Published:** 2021-04-19

**Authors:** Isac Ribeiro Moulaz, Fernanda Sales Soares de Oliveira, Eveline Cristina da Silva, Janine Martins Machado, Maria Carmen Lopes Ferreira Silva Santos

**Affiliations:** 1 Universidade Federal do Espírito Santo, Medical School, Vitória, ES, Brasil; 2 Universidade Federal do Espírito Santo, Medical School, Department of Pathology, Vitória, ES, Brasil; 3 Universidade Federal do Espírito Santo, Medical School, Department of Obstetrics and Gynecology, Vitória, ES, Brasil

**Keywords:** Lactation Disorders, Breast, Breast neoplasms

## Abstract

Lactating adenoma is a rare benign breast lesion that most often presents as a small (up to 3 cm), solid, well-circumscribed, solitary, painless, mobile, lobulated mass. The highest incidence occurs in primiparous women (20 to 40 years old) during the third trimester of pregnancy. However, in the rare case presented herein, in addition to its giant size (more than 10 centimeters on palpation), this lactating adenoma is distinctive due to the presence of multiple nodules, poorly defined ultrasonographic margins, worrisome radiologic features, growth since early pregnancy, presence of infarction and association with chronic mastitis. From the clinical-radiologic perspective, the differential diagnoses included abscess associated with puerperal mastitis, phyllodes tumor, and galactocele. Biopsy was performed, and pathologic examination revealed the classic characteristics of lactating adenoma with multiple infarcted areas, leading to an unexpected confirmed case of giant lactating adenoma.

## INTRODUCTION

Lactating adenoma (LA) is a rare benign breast mass^1^ that usually affects women (20 to 40 years old) during the third trimester of pregnancy or while breastfeeding.^2^ ^,^ ^3^ Although it is most commonly reported in primigravid women, there have been reports in multigravid women. The exact nature of lactating adenoma remains unknown, but some postulate that it may originate as a new lesion from pre-existing adenomas.^3^

Lactating adenoma usually presents as a small (about 3 cm), solid, painless, well-defined, lobulated, mobile nodule. This nodule usually grows slowly and may or may not be palpable.^2^ ^-^ ^5^ Axillary lymph nodes are usually not palpable.

Biopsy is the current gold standard test used to diagnose lactating adenoma and should not be delayed until after delivery.^6^ However, ultrasound (US) and mammography applying the BIRADS classification (Breast Imaging Reporting and Data System) are useful modalities to confirm the need to proceed with tissue sampling.^2^ ^,^ ^5^

## CASE REPORT

A 20-year-old, primiparous woman presented during her third postpartum month with firm right breast enlargement. She was exclusively breastfeeding. She noted that the enlargement began during the first trimester of her pregnancy. On physical examination, a bulky and firm mass in the right upper lateral quadrant was palpated to be more than 10 cm. There were no signs of inflammation in the overlying skin. There was a fistula extruding pasty secretions which were assumed to be from an abscess resulting from puerperal mastitis. Adjacent to the fistular ostium, there was a rubbery mobile mass measuring 3 cm on palpation.

Right breast ultrasonography showed a hypoechoic and heterogeneous mass in the upper lateral quadrant with poorly defined edges measuring 8.8 x 5.9 cm and an adjacent nodule measuring 6.3 x 3.3 cm described as an atypical lymph node enlargement given a BI-RADS 5 classification, which indicates a high suspicion of cancer.

**Figure 1:**
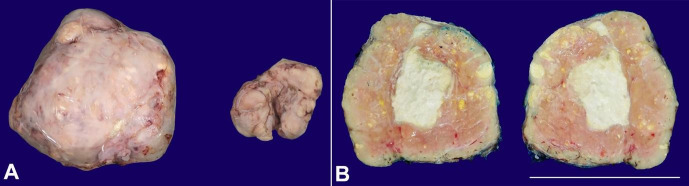
Gross view of the surgical specimens. A – External surface; The larger mass measured 11 x 10 x 6 cm, while the smaller mass measured 3.0 x 4.5 x 2.0 cm. Both were well-defined brown and lobulated nodules with pseudo-capsule. B – Cut section of the larger mass; Heterogeneous solid mass with small peripheral cysts and central, white chalk-like necrotic area (scale bar = 11 cm).

**Figure 2:**
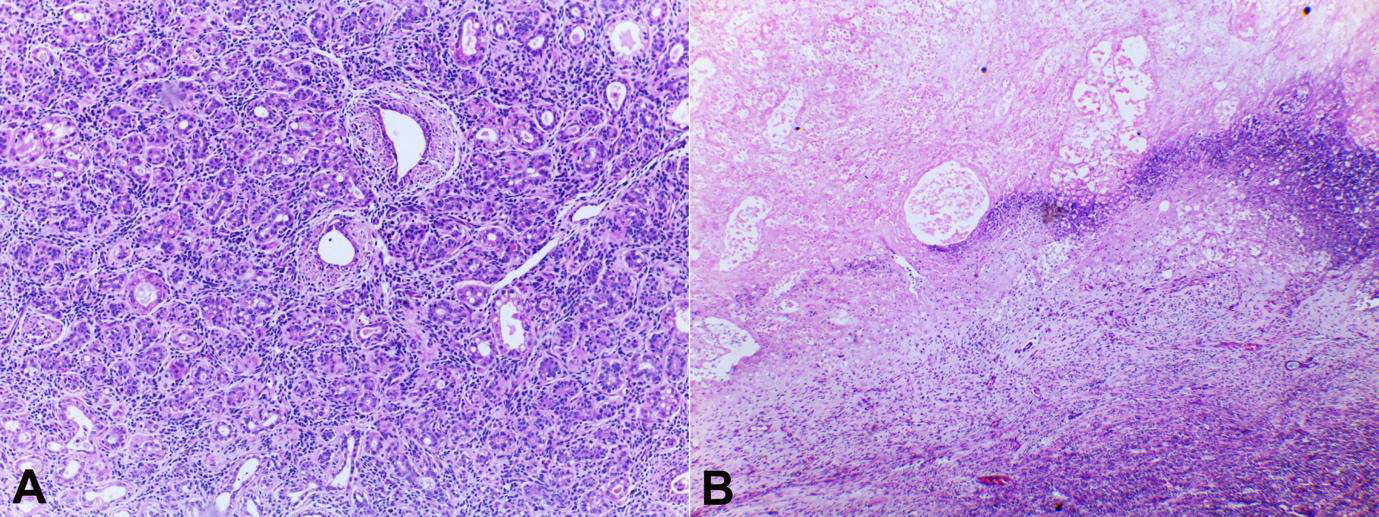
Photomicrograph of the surgical specimen. A – Lactating adenoma; proliferation of intralobular tubules and acini, closely packed and intermixed with delicate fibrous stroma. Some acini with lumina containing eosinophilic secretions (H&E, 40x); B – Microscopic appearance of grossly chalk-like area demonstrating coagulative necrosis and a hyperbasophilic rim of dystrophic calcification (H&E, 40x).

Clinically, the working diagnosis was a phyllodes tumor. Other differential diagnoses included abscess associated with puerperal mastitis and galactocele. Therefore, resection of the lesions was performed. Gross examination of the surgical specimens showed two masses; the larger (Figure 1) weighed 350g and measured 11 x 10 x 6 cm, while the smaller weighed 42g and measured 3.0 x 4.5 x 2.0 cm. Both lesions were well-defined, brown, firm, and rubbery. The cut surface showed a compact and lobulated mass with cystic areas measuring 6.0 x 4.5 cm, containing pasty, friable and sometimes yellow contents, and surface fistulation. The histologic analysis revealed two lactating adenomas (Figure 2), with an area of ischemic infarction and chronic suppurative mastitis in the larger lesion. Two months after surgery, there was a residual 3 cm nodule filled with serosanguinous fluid. Drainage was performed. Outpatient monitoring showed progressive size reduction of the residual nodule and no further complications.

## DISCUSSION

Lactating adenoma is a nodular lesion formed of tubules and acini surrounded by myoepithelial cells and lined by vacuolated epithelial cells, containing abundant intraluminal secretion. It usually spontaneously regresses and is rarely accompanied by axillary lymphadenopathy.^2^ ^,^ ^7^ The typical presentation is a painless, solitary, mobile, well-circumscribed breast mass smaller than 5 cm in diameter. Rarely, they may be multiple or bilateral. It occurs during the third trimester of pregnancy, as well as during breast feeding.^6^ In our case, in addition to its unusually large size (more than 10 cm on palpation), this lactating adenoma presented as multiple nodules. Moreover, this lesion had poorly-defined ultrasonographic margins (BI-RADS 5), its growth began in early pregnancy, and contained areas of necrosis and chronic mastitis.

Lactating adenoma is often a diagnosis of exclusion, after ruling out other benign or malignant causes of a breast mass. In this setting, these entities include fibroadenoma (although lactating adenoma consists of a benign growth of tubules and acini^5^ with scarce stromal epithelial cells), tubular adenoma (which does not show lactational changes such as intracytoplasmic or supranuclear vacuolization),^4^ ^,^ ^8^ carcinoma and breast cancer, among other diseases. It is worth emphasizing that any breast lesion that occurs during lactation should be thoroughly investigated due to the increased risk of carcinomas that metastasize from lactating breasts.^9^ The diagnosis of lactating adenoma, in this case, was not initially expected due to the extremely atypical clinical presentation and unorthodox BI-RADS 5 classification, while the maximum US category reported from lactating adenomas has been BI-RADS 4b.^4^

After the diagnostic biopsy, the treatments range from medication to surgery, depending on the case. Pharmacologic treatments are based on prolactin inhibitors such as bromocriptine^3^ ^,^ ^10^ and cabergoline,^4^ and tend to have a favorable response. However, the use of anti-prolactin therapy should be weighed with the patient's desire to breastfeed. Most cases of lactating adenoma spontaneously resolve; nonetheless, if it persists, grows rapidly, and/or produces marked discomfort, surgical removal is recommended and can be done during pregnancy without harm to the mother nor fetus. Surgical removal does not impair breastfeeding.^3^ ^,^ ^5^ However, if possible, excision should be postponed to the postpartum period. The patient should be followed-up within a few months to exclude complications or relapse.

## CONCLUSION

We present a rare case of lactating adenoma which was highly suspicious for malignancy at clinical presentation and ultrasonography. The dominant mass with satellite nodules, necrotic center, ill-defined borders and related lymphadenopathy were interpreted as BI-RADS 5 category on ultrasonography. Biopsy unexpectedly revealed benign-appearing lactating adenoma. The presence of multiple nodules, inflammatory edema, and necrosis due to ischemic central area of rapid enlarging mass contributed to the high suspicion of malignancy before biopsy was performed.

## References

[1] Lakhani S, Ellis I, Schnitt S (2012). WHO Classification of Tumours of the Breast..

[2] Kumar H, Narasimha A, Bhaskaran MN, M N DR (2015). Concurrent lactating adenoma and infiltrating ductal carcinoma: a case report. J Clin Diagn Res.

[3] Teng CY, Diego EJ (2016). Case report of a large lactating adenoma with rapid antepartum enlargement. Int J Surg Case Rep.

[4] Elzahaby IA, Saleh S, Metwally IH, Fathi A, Atallah K (2017). Huge lactating adenoma of the breast: case report. Breast Dis.

[5] Barco Nebreda I, Vidal MC, Fraile M (2016). Lactating Adenoma of the breast. J Hum Lact.

[6] Szabo J, Garcia D, Ciomek N, Margolies L (2017). Spuriously aggressive features of a lactating adenoma prompting repeated biopsies. Radiol Case Rep.

[7] James K, Bridger J, Anthony PP (1988). Breast tumour of pregnancy (‘lactating’ adenoma). J Pathol.

[8] Manipadam MT, Jacob A, Rajnikanth J (2010). Giant lactating adenoma of the breast. J Surg Case Rep.

[9] Schmitt FCDL, Gobbi H, Filho GB (2016). Mama. Bogliolo Pathology.

[10] Reeves ME, Tabuenca A (2000). Lactating adenoma presenting as a giant breast mass. Surgery.

